# Barriers to utilization of postnatal care at village level in Klaten district, central Java Province, Indonesia

**DOI:** 10.1186/s12913-017-2490-y

**Published:** 2017-08-07

**Authors:** Ari Probandari, Akhda Arcita, Kothijah Kothijah, Eti Poncorini Pamungkasari

**Affiliations:** 1 Department of Public Health, Faculty of Medicine, Universitas Sebelas Maret, Jl. Ir. Sutami 36A, Surakarta, 57126 Indonesia; 2 Master Program of Public Health, Graduate School, Universitas Sebelas Maret, Surakarta, Indonesia; 3 Bethesda Health Institute, Yogyakarta, Indonesia; 4 Vocational Program of Occupational Health and Safety, Faculty of Medicine, Universitas Sebelas Maret, Surakarta, Indonesia

**Keywords:** Postnatal care, Patient-centered care, Midwives, Maternal health, Continuity of care, Indonesia

## Abstract

**Background:**

Maternal health remains a persisting public health challenge in Indonesia. Postnatal complications, in particular, are considered as maternal health problems priority that should be addressed. Conducting adequate care for postnatal complications will improve the quality of life of mothers and babies. With the universal health coverage implementation, the Indonesian government provides free maternal and child health services close to clients at the village level, which include postnatal care. Our study aimed to explore barriers to utilization of postnatal care at the village level in Klaten district, Central Java Province, Indonesia.

**Methods:**

A qualitative study was conducted in March 2015 – June 2016 in Klaten district, Central Java, Indonesia. We selected a total of 19 study participants, including eight mothers with postnatal complications, six family members, and five village midwives for in-depth interviews. We conducted a content analysis technique on verbatim transcripts of the interviews using open code software.

**Results:**

This study found three categories of barriers to postnatal care utilization in villages: mother and family members’ health literacy on postnatal care, sociocultural beliefs and practices, and health service responses. Most mothers did not have adequate knowledge and skills regarding postnatal care that reflected how they lacked awareness and practice of postnatal care. Inter-generational norms and myths hindered mothers from utilizing postnatal care and from having adequate nutritional intake during the postnatal period. Mothers and family members conducted unsafe self-treatment to address perceived minor postnatal complication. Furthermore, social power from extended family influenced the postnatal care health literacy for mother and family members. Postnatal care in the village lacked patient-centered care practices. Additionally, midwives’ workloads and capacities to conduct postnatal information, education and counseling were also issues.

**Conclusions:**

Despite the government’s efforts to provide free postnatal care closer to mothers’ homes, other barriers to postnatal care utilization remained. Specifically, among mothers, community, and health services. An innovative approach to increase the health literacy on postnatal care is required. In particular, improving the capacity of midwives to conduct patient-centered care. In addition, village midwives’ tasks should be evaluated and reoriented.

**Electronic supplementary material:**

The online version of this article (doi:10.1186/s12913-017-2490-y) contains supplementary material, which is available to authorized users.

## Background

Maternal health in Indonesia is a persisting public health challenge. Indonesia is one of ten countries that contribute to 59% of the global maternal deaths. While progress was observed in meeting the Millennium Development Goals (MDGs) to reduce the Maternal Mortality Ratio (MMR), other South-East Asian countries have shown faster progression [1–3].

Postnatal complications are considered as important maternal health problems that should be addressed. During the first postnatal year, postnatal complications could increase the risk to perinatal and infant mortality and other mental problem. The complications also imply negative financial and productivity consequences [4, 5].

In Indonesia, the postnatal service utilization is lower than the skilled birth attendance coverage [6]. Additionally, postnatal care utilization in the rural area of Indonesia is lower than in the urban area, similar to other low- and middle-income countries [7].

In the current era of universal health coverage implementation, Indonesia has put efforts into implementing a large-scale village midwives program and provides free maternal-and-child health services that are closely located to clients at the village level [2, 8]. These efforts include provision of the standard four postnatal care sessions, i.e. on the first day, the 6th day, the 14th day, and the 6th weeks after childbirth. The standard postnatal care covers physical examinations (of vital signs, breast, the fundus of the uterus, lochia and other vaginal discharges), communication/information/education on exclusive breastfeeding and family planning. A home visit for postnatal care by the midwives should be conducted when mothers do not visit the village clinic [9].

Central Java province is one of the provinces with high maternal mortality in Indonesia [6]. Klaten district is a rural district in Central Java province that reported an increase MMR during 2011-2013, from 54 to 118 per 100,000 live births. Furthermore, the maternal mortality in the district has not shown a significant improvement with 116 deaths per 100,000 live births in 2014. Approximately 70% of the maternal mortality cases in 2014 occurred during the postpartum period [10].

Previous studies on postnatal care utilization in Indonesia were conducted before the launch of the national health insurance policy in 2014 [11, 12]. Those studies highlighted problems on access to postnatal care. Despite efforts of the Indonesia government to improve financial and physical access to maternal health care, utilization of postnatal care is still suboptimum. Evidence is lacking on the barriers of postnatal care utilization in the new context of the national health insurance policy implementation, particularly in rural Indonesia. Hence, our study aimed to explore barriers to utilization of postnatal care at the village level in Klaten district, Central Java Province, Indonesia.

## Methods

### Research setting

The present study was conducted in Klaten district, Central Java Province, Indonesia from March 2015 to June 2016. In that year, 1,130,047 inhabitants were distributed among 26 subdistricts. Approximately 54.28% of the population was living in rural areas. The literacy rate among people aged ≥15 years old was 88.73%. The proportion of the population who had reached at least a 9-year basic education level was 43.54% [13].

### Study design

We conducted a qualitative study with a phenomenological approach that emphasized on study participants’ subjective experience of a phenomenon [14]. This approach was appropriate to address our study aim: exploring the barriers to postnatal care utilization in the villages from the perspectives of the mothers, family members, and village midwives.

### Researcher characteristics and reflexivity

AP and EPP have experiences in conducting qualitative research. AA has a professional background as a midwife and is working in a nursing school. K is experienced in community engagement. All authors are familiar with the context of the study area and accustomed to communicate using Javanese language. We built rapport with the study participants by introducing our positions as researchers and conducting informed consent carefully.

### Study informants and sampling

We selected mothers with postnatal complications by criterion sampling based on the maternal and child health program register in Klaten district health department. Mothers with postnatal complications were selected due to the significance of utilizing postnatal care for these mothers. We also included family members who were involved in taking care of the selected mothers during postnatal period. Therefore, we could explore their influence to the mother’s utilization of postnatal care. We also interviewed village midwives who worked in the village where the selected mothers live, who were responsible to provide postnatal care to the mothers.

### Data collection

Data were collected via in-depth interviews by AP and AA. For mothers and family members, the interviews were conducted at the participants’ home. For midwives, the interviews occurred at the village maternal and child clinics. We also reviewed the medical records of the mothers to confirm the frequency of postnatal visits they attended. We offered the participants to conduct the interviews in Javanese or Bahasa Indonesia language. The interview guide (see the Additional file 1) covered topics on:

- knowledge on postnatal period
- utilization of postnatal care (how many, when, where)
- content of postnatal care received (e.g. physical examination, education/counseling)
- the role of the family during the postnatal period
- beliefs and traditional practice related to the postnatal period.

### Data processing and analysis

All interviews were audio recorded and written for the verbatim transcripts. Directed content analysis was conducted to evaluate the data [15], by using Andersen’s and Newman’s framework of determinants of health services utilization i.e. societal determinant (including norm of the community), health services system and individual determinant [16].

We used open code software to assist in extracting meaning units, codes, and categories from the verbatim transcript [17]. AP and AA conducted coding independently. A consensus was reached after results were further discussed with K and EPP. Examples of the coding process are presented in Table 1.

**Table 1:** Examples of coding processes

Meaning Unit	Code	Sub-category	Category
I visited the midwife’s clinic only after I got infection, not before that (Mother 8).	Knowledge on danger signs	Knowledge	Mother and family members’ health literacy
Many mothers believe in food taboo...They are also afraid to do self-wound care...(Midwife 1).	Attitude to food taboo Lacking confidence to do self-wound care	Awareness and confidence	
The midwife gave the advice to visit the village health clinic before 7 days, but I did it after 10 days (Mother 6).	Delay to conduct postnatal care visit	Practice	
I informed my daughters about the traditional rules. She is submissive to my suggestions. She should not eat peanuts and fish if her wound has not dry enough unless she will get an infection. When I delivered my daughter a long time ago, I eat fish and then my daughter’s cord got infection (Family 1).	Feeling afraid to against parents/grandmothers’ advice	Social power	Social power, cultural belief and practices
	Food taboo	Cultural belief and practice	
I am aware that it should be home visit but in case that I felt that the patients visited me at the clinic, I perceived that that was a home visit (Midwife 3).	Inconsistent home visit	Provider work load and capacity	Health service responses
I received only wound care...Maybe because my condition was good. My blood pressure was high only when I delivered the baby, after that my blood pressure was normal (Mother 7).	Selective care	Perceived low quality of postnatal care	
I did not understand what the the widwife said. She said some sentences in the Javanese language, the high-level Javanese level that I did not really understand (Mother 1).	Language barrier	Suboptimal patient-centered care	

### Trustworthiness

We triangulated the information gathered from the mothers, family members, and village midwives to increase the validity of our results. Peer debriefing between all authors was conducted to discuss the codes and categories generated from the data.

### Research ethics

Written individual informed consent was sought from the study participants, including their consent to record the interviews. An administrative procedure was followed before we assessed the mothers’ medical records. Informant identity during data analysis and reporting were kept confidential. This study received ethical clearance from the Ethics Committee, Faculty of Medicine, Universitas Sebelas Maret, Surakarta, Indonesia.

## Results

Eight mothers, six family members, and five village midwives (19 in total) participated in this study. Most mothers experienced severe postnatal complications (postnatal hemorrhage and pre-eclampsia), while few mothers suffered from infection, chronic energy deficiency and anemia.

Our analysis revealed three categories of barriers to postnatal care utilization at the village level: [1] mother and family member’s health literacy on postnatal care, [2] social power, cultural belief and practices, and [3] health service responses. Table 2 presents overall codes that constitute the categories.

**Table 2:** Synthesis of data: coding and categories

Categories	Mother and family members’ health literacy			Social power, cultural belief and practices		Health service response		
Sub-categories	Knowledge	Practice	Awareness and confidence	Social power	Cultural belief and practices	Working load and capacity of midwives	Perceived low quality of postnatal care	Suboptimal patient-centered care
Codes	Knowledge on postnatal period; Knowledge of danger signs; Lack of knowledge on safe self-treatment.	Lack of hygiene; Delay to conduct postnatal care visit; Lack skills to do wound-care.	Lack of confidence to do self-wound care; Attitude to food taboo; Submissive to the midwife’s advice.	Information from family; Information from neighbors; Information from parents; Submissive to what parents said; Feeling afraid of parents/ grandmothers; Feeling afraid to disobey parents/grandmothers’ advice; Living with parents.	Traditional rules; Intergeneration practice; Cultural self-treatment practice; Practices from grandmothers; Food taboo; Herbal potion; Breastfeeding booster potion; Unseen hazards.	Working load; Collaboration between village midwives and health cadres; Knowledge of midwives; Education on food taboo.	Timely service; Availability of midwives; Insuficient time to educate patients; Lack of education on breastfeeding; No information for next visit; No education to family; Perceived quality of service; Inconsistent home-visit; Selective care; Inadequate education for wound care; Indirect communication between midwives and mothers.	Lack of interactive communication; Lack of frank communication; Language barrier; Inconvenient treatment.

### Mother and family member’s health literacy on postnatal care

Our study found the lack of knowledge, awareness, and practice related to postnatal care. Most mothers and family members did not know about the characteristics and duration of the postnatal period, as expressed by one of the informants: “I got a lot of bleeding. I did not know whether that was normal or not. I received no information about that… I experienced bleeding after 40 days. There were bleeding spots, but I do not know whether it was normal or not.” (Mother 3, multipara, 35 years old).

Most mothers in our study did not recognize their needs during the postnatal period, particularly on nutrition, lactation and self-hygiene. Information and education about postnatal care were lacking, and there were misconceptions and myths among mothers and family members (see the section of sociocultural beliefs and practice).

Mothers lacked skills in performing postnatal hygiene care. “Mothers are afraid to care for the [episiotomy or cesarean surgery] wound. While I have given information that every time they go to toilets they should change sanitary napkins and do the wound care.” (Midwife 1, 38 years old).

Most mothers received only one session of postnatal care during some hours/days at the health facility. They did not adhere to the next postnatal care schedule unless they (or the newborn) experienced health problems. Meanwhile, midwives reported insufficient time to conduct the home visits to mothers when the mother does not come to the village clinic (see the section of health services response).

“I did a health check up once at the hospital. I also went to our local midwife’s practice twice. I did the first visit because I got a headache. I did the second visit when my kid got diarrhea.” (Mother 1, primipara, 26 years old).

“... A week after the delivery I was suggested to check up to the doctor at the health center. But I missed that. I forgot.” (Mother 2, multipara, 40 years old).

### Sociocultural beliefs and practices

#### Culture-related myths and rules

Several myths on postnatal care in the study area persisted. One of the myths was, “A women who just delivered a baby could not go out far from the house. My parents said that it is a prevention of the mother and her baby to get any unseen hazard.” (Family member 2, female, 45 years old).

The lack of knowledge on good nutritional intake during postnatal care was related to the presence of food myths in the area. Mothers were expected to obey traditional rules such as not eating fried food, chicken, egg white, fish, peanuts, and some vegetables with high protein. These types of food were perceived to delay the episiotomy or cesarean surgery wound healing.

" ... I must not eat fish. I must not eat chicken and salted fish.... I am afraid to get itchy on my stitches. My neighbors said that it would make my stitches worse." (Mother 4, primipara, 23 years old)

“There is a traditional rule that during postnatal period mothers should only eat green vegetables, no chili, not fried, no meats. Mothers should limit the amount of water to drink because it will make their babies have a cold.” (Midwife 5, 46 years old)

There was a belief among most mothers and family members that consuming animal products would negatively affect mothers and their babies. “I should limit of eating any fish. If I eat fish, my breast milk will be fishy.” (Mother 1, primipara, 26 years old).

#### Self-treatment and traditional care

Our study revealed that existing self-treatment and traditional care practice in the rural community might influence postnatal care utilization at the village clinics. For example, one mother who suffered from an infected episiotomy wound bought antibiotic pills from a pharmacy for self-treatment. She mixed the pill contents with vegetable oil and placed this mixture on top of the wound. The family of the mother stated, “This is the medicine since a long time for any types of wounds.” (Family member 1, female, 68 years old).

There were existing traditional practices to improve the fitness of mothers and to improve the production of breast milk. One mother stated, “I drank herb potion, to facilitate breastfeeding.” (Mother 5, multipara, 30 years old). A family-member informant confirmed about the tradition. “The Javanese people used the traditional herb to improve the production of breast milk.” (Family member 6, female, 45 years old).

#### Social power

Culture-related myths and rules were passed on by parents, parents-in-law and other elder extended families to the mothers. Mothers who lived in the same house with their parents, parents-in-law or grandparents were more inclined to follow such myths and rules.

“I do not know the reasons for the rules. I just obey my parents.” (Family member 1, female, 72 years old)

“Culture-related myths and rules still exist among a small proportion of population particularly among pregnant women and mothers who lived with their parents and grandparents. I discussed this issue in the education class for pregnant women. However, it is difficult to change the knowledge of mothers who live with grandparents. They do not believe it, but they are afraid to violate the rules from their parents.” (Midwife 2, 32 years old).

### Health services responses

#### Perceived low quality of postnatal care

Several mothers and family members perceived health services in the village clinics as being of low quality. This was reflected in their preference to visit a hospital or private midwives for postnatal care instead of utilizing the free postnatal care at the village. We also revealed the mistrust toward village midwives’ abilities. One mother stated, “The midwife is still young; I guess she lacks experience” (Mother 2, multipara, 40 years old). Some mothers also complained about village clinics’ unreliable service hours.

Most of the mothers attended only the first postnatal care service. The postnatal care was not continued unless midwives informed the mothers that there were persisting health problems. Triangulated document reviews showed that most mothers attended only one postnatal care visit during the postpartum period.

"After 1 week, I did check myself up. After that, I did not consult again. I thought once was enough. I was suggested to have my stitches checked if necessary ... I did go to the midwife because I got a headache, one month after delivery." (Mother 1, primipara, 26 years old)

During home visits, midwives conducted selected physical examinations which were only relevant to the health complaints. Most mothers reported receiving no information, education, and counseling on postnatal care. "She checked me on here [she pointed her chest], then she checked out my stitches. No other information. Blood pressure was checked." (Mother 3, multipara, 35 years old).

There were variations in the information and education provided by the village midwives. Information and education about medicine that mothers should take were given, but only few midwives delivered messages on hygiene, breast care and breastfeeding. The majority of the midwives did not give any education to the mothers on how to keep the hygiene of cesarean surgery or episiotomy wound. The midwives gave topical antiseptic lotion and explained how to apply it to the mothers. However, they did not evaluate whether the mothers were confident and able to perform cesarean surgery or episiotomy wound care. Few midwives gave counseling to the mother and husband about the importance of family support.

#### Suboptimal patient-centered care

The process of patient education at village clinics lacked a patient-centered care principle. One mother stated that the midwife gave health information about postnatal care immediately after delivery, when she was still under pain. A process to ensure that patients understand the information provided by midwives was suboptimal. The midwife delivered the information using high-manner Javanese language, which was not understood by all Javanese people. Another mother experienced uncomfortable treatment when the midwife removed stitches from the post-cesarean section. “The midwife took out the stitches with pressure. I was shocked.” (Mother 8, primipara, 38 years old).

There was a perceived lack of trust toward village midwives. One-direction and ineffective communication often occurred between the midwife and mother. The mothers did not always convey information honestly. One midwife illustrated, “The problem is that mothers could not say their problems… About exclusive breastfeeding, in front of me they said they gave breastfeeding to their baby, but when I cross-checked the information with others, I knew that the mothers used formula milk.” (Midwife 1, 38 years old).

#### Workload and capacity of midwives

The working load of midwives was another bottleneck in performing postnatal care at village clinics. In addition to providing perinatal care at village maternal and child clinics, they were also expected to run other mother and child health programs.

“I know that I should conduct a home visit for pregnant women, do postnatal care, and monitor high-risk neonates. However, the population which I should serve is too large. There are 500 under five children, more than 100 pregnant women per year, 114 infants... I also have other tasks for dengue control program, helminthiasis control program... I have limitations to conducting a home visit. I am aware that my works are suboptimal.” (Midwife 3, 43 years old)

Providing the standard postnatal care including home visits was also difficult for midwives who did not live in the village. Only one of five midwives in our study mentioned collaboration with health cadres and/or private midwives in the village to overcome her limited capacity to conduct postpartum monitoring.

## Discussion

Our study highlighted findings on barriers related to the utilization of postnatal care at village clinics: community’s literacy on postnatal period and care, sociocultural belief and practices, and health service responses. These findings signifies the importance of overcoming those three barriers beyond the efforts to improve financial and physical access to postnatal care.

### Community’s literacy on postnatal period and care

Most of the mothers in our study lacked health literacy including knowledge, skills, and awareness of the postnatal period. Another study in West Java, Indonesia also highlighted the problem of awareness on postnatal care [11]. Other studies also reported the very low proportion of mothers with a sufficient level of knowledge and awareness about the danger signs during pre- [18] and post-natal care [19].

### Sociocultural beliefs and practices

Our study provides insight into the low utilization of village clinics’ postnatal care. Another study in Nepal found that mothers in rural areas utilized postnatal care less than mothers in urban areas [20]. The low utilization of postnatal care in the rural area may be related to existing sociocultural beliefs and practices, which also persist in rural Indonesia. We found that the traditional practice of taking herbal potion was prominent among mothers during pregnancy and after delivery, which is consistent with findings from previous studies [21–23]. The traditional care has been upheld by the community for many years to address postnatal problems.

We highlighted the role of social power, not only of the immediate family members, but also the extended family and neighbors. They influence the knowledge, attitude, and practices of mothers during the postnatal period. In our study, mothers who lived with their parents and/or extended family had less adherence to midwives’ health education. A study in Pakistan showed that mothers first turned to close relatives when searching for information and help regarding postnatal illness [24]. A study in Ethiopia highlights the level of decision-making authority as the determining factor for postnatal care utilization [25].

### Health service responses

Based on the World Health Organization’s (WHO) recommendation, the government of Indonesia develop a guideline aims to provide four times of postnatal care to all mothers and neonates during the first 6 weeks after birth. However, most mothers in our study only received one to two postnatal care visits. The WHO guideline on postnatal care emphasizes that home visits can be conducted by midwives or well-trained and supervised community health cadres. Our study revealed challenges to implementing this guideline due to the midwives perceived work burden. Specifically, because the village midwives were responsible to other public health programs in addition to maternal and child healthcare. Therefore, improving the collaboration between village midwives and community health cadres became crucial to overcome these challenges. Additionally, intervention should also focus on training and transportation support to conduct home visits, as a study in China found the lack of staff and transportation for home visits [19].

Our study also showed insufficient quality of services and communication skills to conduct patient-centered care, which was shown to be a determinant for postnatal care utilization in a previous study in Malawi [26]. In addition, counseling about postnatal problems was insufficient and lacked empathy. A study in Ghana revealed issues of communication between health workers and mothers. Specifically, insufficient information on the postnatal care that were performed by the healthcare provider [27]. Nevertheless, mothers expressed their desire to discuss their experience during childbirth and challenges during postnatal care [28].

In Indonesia and other low- and middle-income countries, there could be similarities and differences on the barriers to postnatal care utilization with that of antenatal care. For example, the lack of knowledge among mothers are common for both antenatal and postnatal care [29]. However, low education level and financial barriers were the more predominant factors for utilization of antenatal care [29, 30]. Whereas for postnatal care utilization, cultural related myths and rules, as well as the power of family members like mother or mother-in-law, are the more predominant barriers, as observed in our study and other [31]. Meanwhile, the influence of husband in decision making is more relevant to antenatal care utilization [32].

### Implications for public health practice

Our findings suggest that mothers and their families should receive adequate and continuous information on postnatal care. Mothers’ skills and confidence to perform self-management safely during postnatal periods should also be improved. Furthermore, innovative approaches and novel communication strategies should be implemented to improve the community’s literacy on postnatal care. Mobile Health (mHealth) technology could potentially be used to fill the information gap particularly for mother and child health care services [33–36]. It is particularly relevant in Indonesia, considering that 85% of Indonesians own mobile phones [37]. To support the contextual adaptation of innovations related to postnatal care, an implementation research is needed [38].

A context-specific approach is required to change the irrational social beliefs and practices related to postnatal care. These behavior changes could be achieved by involving extended family and community leaders. Intervention to address these sociocultural issues should include counseling, information, and education for mothers and extended family, using appropriate language. Furthermore, maternal health care providers should be trained to deliver counseling, information, and education while considering the sociocultural context. This context-specific intervention was shown effective to reduce maternal mortality [39].

In addition to improving access, we agree with others that actions addressing the quality of maternal health care are needed [40]. Our study showed the variation and insufficiency in postnatal care provision. The lack of a responsive postnatal service to mothers’ needs suggests the urgency to revisit the current curriculum for village midwives training. Indonesia has provided a national guideline on the continuum of care from pregnancy to postnatal care [41] that should be combined with hands-on skills training for front-line providers. These steps should also be followed by supportive supervision of the implementation.

### Limitations and strengths of the study

This study focused on the exploration of postnatal care barriers at public village clinics in Klaten, Central Java, with relatively accessible healthcare facility. Additionally, our study was conducted in the context of universal health coverage policy. Therefore, geographical and financial barriers reported in other studies in low- and middle-income countries [12, 19], were not identified in our findings. However, our study revealed the barriers to postnatal care service utilization in the era of universal health coverage; that has not explored in-depth in previous studies [11, 12]. The findings from our study could be generalized in other population with similar sociocultural setting, in Indonesia and other developing countries.

## Conclusion

Our study concluded that despite efforts to provide free postnatal care closer to mothers’ homes, important barriers to its utilization persist. Specifically, community health literacy on postnatal care, sociocultural beliefs and practices, and health service responses. Community health literacy on postnatal care should be improved through innovative information, education and counseling programs targeting mothers and families. Community engagement is essential to address any unsafe and irrational sociocultural beliefs and practices. Postnatal care quality should be improved by upgrading the skills of midwives to deliver patient-centered care. Innovative interventions and implementation research should be combined to improve postnatal care utilization in Indonesia.

## Additional file

Additional file 1: The guideline for the in-depth interviews with mothers, the family members, and midwives. (DOCX 60 kb)
