# Supplementary material for: Barriers to utilization of postnatal care at village level in Klaten district, central Java Province, Indonesia
Source: BMC Health Serv Res. 2017 Aug 7;17:541. doi: 10.1186/s12913-017-2490-y (PMC5547562; doi:10.1186/s12913-017-2490-y)
Supplement: Additional file 1: — The guideline for the in-depth interviews with mothers, the family members, and midwives. (DOCX 60 kb) [file 12913_2017_2490_MOESM1_ESM.docx]

**File Name**: The guideline for interview

**Title**: The guideline for interview (mothers)

**Description**: This was the guideline for interview the mothers with postpartum complication

1. Introduction

- An introduction about the interviewer
- Information about the study

2. Informed consent process

- Explanation about the objective about the interview and time duration needed
- Explanation about rights of the study informants in relation to study participation
- Permission to record the interview
- Consent to the study

3. Record the identity of the study informant

- Name
- Age
- Ethnic
- Education
- Occupation
- Date of her baby delivery
- Place of her baby delivery
- Postpartum complication

4. Questions

1. What do you know about the postpartum period?

Probing: duration, normal and abnormal physical signs can be happened during the postpartum period

1. Do you visit the village maternal and child clinic after your delivery?

Probing: when, frequency, why, kind of services received

1. Do you receive any home-visit from the village midwife during your postpartum period?

Probing: when, frequency, kind of services received (vital sign, health education/counseling)

1. How do you think about the support of family during the postpartum period?
   Probing: support from husband, other family member who live in the same house, neighbors, village midwives
2. Are there some traditional beliefs related to postpartum that you recognize? What do you perceive about that?

5. Closing

- Say thanks
- Leave the contact number of the researcher

**Title**: The guideline for interview (family)

**Description**: This is the guideline for interview with the family-member of the mother with postpartum complication

1. Introduction

- An introduction about the interviewer
- Information about the study

2. Informed consent process

- Explanation about the objective about the interview and time duration needed
- Explanation about rights of the study informants in relation to study participation
- Permission to record the interview
- Consent to the study

3. Record the identity of the study informant

- Name
- Age
- Ethnic
- Education
- Occupation

4. Questions

1. What do you know about the postpartum period?

Probing: duration, normal and abnormal physical signs can be happened during the postpartum period

1. How do you think about the support of family during the postpartum period?
   Probing: support from husband, other family member who live in the same house, neighbors, village midwives
2. Are there some traditional beliefs related to postpartum that you recognize? What do you perceive about that?

5. Closing

- Say thanks
- Leave the contact number of the researcher

**Title**: The guideline for interview (midwife)

**Description**: This is the guideline for interview with the village midwife who work for the area of the mother has been living

1. Introduction

- An introduction about the interviewer
- Information about the study

2. Informed consent process

- Explanation about the objective about the interview and time duration needed
- Explanation about rights of the study informants in relation to study participation
- Permission to record the interview
- Consent to the study

3. Record the identity of the study informant

- Name
- Age
- Ethnic
- Education

4. Questions

1. Are there some traditional beliefs related to postpartum that you recognize? What do you perceive about that?
2. How do you think about the support of family during the postpartum period?
   Probing: support from husband, other family member who live in the same house, neighbors.
3. How do you perceive about the implementation of postpartum services in the village level?

Probing: when, frequency, kind of services, home-visit, barriers of the implementation

5. Closing

- Say thanks
- Leave the contact number of the researcher
